# Highly synergistic antimicrobial activity of magainin 2 and PGLa peptides is rooted in the formation of supramolecular complexes with lipids

**DOI:** 10.1038/s41598-020-68416-1

**Published:** 2020-07-15

**Authors:** Christopher Aisenbrey, Mariana Amaro, Petr Pospíšil, Martin Hof, Burkhard Bechinger

**Affiliations:** 1 Institut de Chimie UMR7177, CNRS, University of Strasbourg, 1, rue Blaise Pascal, 67000 Strasbourg, France; 2 J. Heyrovský Institute of Physical Chemistry, v.v.i, Czech Academy of Sciences, Dolejškova 2155/3, 182 23 Prague, Czech Republic; 3 Institut Universitaire de France, Paris, France; 4 Present Address: Institut für Angewandte Physik and Center for Functional Nanostructures (CFN), Karlsruhe Institute of Technology, Wolfgang-Gaede-Straße 1, 76131 Karlsruhe, Germany

**Keywords:** Solid-state NMR, Biological fluorescence, Membrane biophysics, Membrane structure and assembly

## Abstract

Magainin 2 and PGLa are cationic, amphipathic antimicrobial peptides which when added as equimolar mixture exhibit a pronounced synergism in both their antibacterial and pore-forming activities. Here we show for the first time that the peptides assemble into defined supramolecular structures along the membrane interface. The resulting mesophases are quantitatively described by state-of-the art fluorescence self-quenching and correlation spectroscopies. Notably, the synergistic behavior of magainin 2 and PGLa correlates with the formation of hetero-domains and an order-of-magnitude increased membrane affinity of both peptides. Enhanced membrane association of the peptide mixture is only observed in the presence of phophatidylethanolamines but not of phosphatidylcholines, lipids that dominate bacterial and eukaryotic membranes, respectively. Thereby the increased membrane-affinity of the peptide mixtures not only explains their synergistic antimicrobial activity, but at the same time provides a new concept to increase the therapeutic window of combinatorial drugs.

## Introduction

Magainin 2^1,2^ and PGLa^3,4^ are stored together in the skin of *Xenopus laevis* frogs where they constitute an important part of the innate immune system^5^. When added together the two peptides show an order of magnitude synergistic enhancement in their antibiotic activities^6–8^ as well as in biophysical assays^7^. Understanding the mechanisms of action of antibiotic peptides and their synergistic or super-additive behavior is of special interest since it helps to guide the development of more potent antibiotics. Many efforts have been made to elucidate the structure of a possible complex of magainin 2 and PGLa^8–11^ and to correlate those structural features with function. The formation of pores by a barrel stave mode of action^12^ is the oldest model for antibiotic activity and very intuitive. It leads to the “search” for transmembrane configurations, which can be found for PGLa when reconstituted into fully saturated artificial model membranes^13,14^. However, most cationic amphipathic antibiotic peptides adopt stable alignments parallel to the membrane surface and lack the transmembrane configuration that is a hallmark of the barrel-stave model^15–17^. An instructive example is the antibiotic designer peptide LAH4, which adopts an in-planar configuration at low pH and is transmembrane at high pH^18^. In contrast to what is predicted from a barrel stave model the antibiotic activity of LAH4 is increased at low pH, where the peptide is oriented parallel to the membrane surface^19^. This and related observations have led to new concepts such as the carpet^20^ or the detergent models^21^. More recently, based on these prior ideas the SMART model has been introduced^22^ which expands our views much beyond the picture of a pore formed by a transmembrane bundle of helical peptides^23^. Common to all models is the formation of supramolecular complexes made from antimicrobial peptides and lipids. Thereby causing the membranes to rearrange in shape and packing the peptides tend to disrupt the integrity of the lipid bilayer.

Notably the influence of magainin 2 on the alignment of PGLa within the membrane depends on the saturation of the lipids used for the experiments^14,24,25^. In addition, the synergistic behavior of magainin 2 and PGLa has been shown to depend on the intrinsic curvature of the bilayer^26^. Considering the high content of phosphoethanolamine (PE) in bacterial membranes^27^, POPE/POPG bilayers represent a good model to investigate antibacterial activities by biophysical approaches. Both findings are indicators of an important role of the lipids that goes beyond the idea that they merely constitute a two dimensional liquid which accommodates active protein components^28^. Lipids can mediate peptide interactions^24^ or form lipid/peptide supercomplexes^29^. Direct peptide-peptide interactions, such as salt bridges/Coulomb interactions or in-membrane GxxxG dimerization motives^30^, might also contribute to the formation of complexes^31^.

Therefore, here we focus on the characterization of the supramolecular complexes without assumptions about the existing models for antibiotic activity. Highly dynamic supramolecular complexes are difficult to grasp, but fluorescence correlation spectroscopy (FCS) and fluorescence self-quenching techniques^32^ allow the characterization of the nanoscale environment surrounding the peptides. Beyond structural aspects, we investigate the thermodynamics of the interaction of magainin 2, PGLa and lipid bilayers. Furthermore, a quantitative description of the packing and clustering of peptide molecules at the membrane surface is obtained. Finally, the findings are put into perspective relative to different models previously developed for antibiotic action^20^.

## Results

Titrations of magainin 2 and PGLa in presence of GUVs (POPE/POPG, 2/1 mol/mol) were performed in combination with z-scan FCS. The FCS technique is sensitive to the diffusion of the (labeled) magainin 2 and PGLa peptides on the surface of the GUVs and allows to quantify the surface concentration of the moving particles (peptides). For such experiments magainin 2 was labeled with BODIPY 650/665-X (Mag2-r) and PGLa was labeled with FluoProbes FL SE (PGLa-g), two chromophores with distinct emission spectra and no significant FRET. The measurements were performed above the gel-to-liquid crystalline phase transition of this lipid mixture, which occurs at about 21 °C^27^.

**Figure 1:**
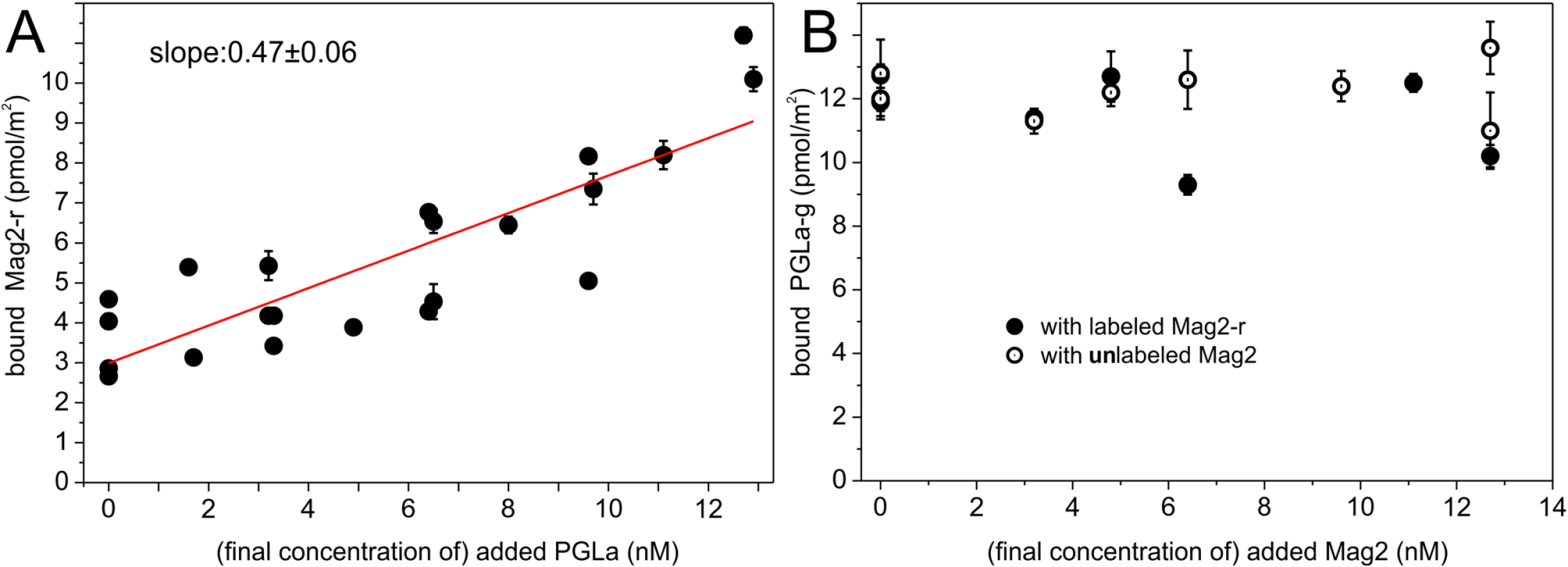
(**A**) Surface concentration (pmol/m^2^) of bilayer-associated red particles (i.e. diffusing particles of Mag2-r) as a function of the solution concentration (nM) of unlabeled PGLa. The Mag2-r concentration in solution is (approximately) constant at 13 nM. (**B**) Surface concentration (pmol/m^2^) of bilayer-associated green particles (i.e. diffusing particles of PGLa-g) as a function of the solution concentration (nM) of magainin 2 (labeled or unlabeled). The PGLa-g concentration in solution is (approximately) constant at 13 nM. Experiments were performed at 24 °C.

The surface concentration of membrane-bound Mag2-r was quantified in the presence of increasing amounts of PGLa. The samples consisted of approximately 16 nmol (58 µM) of lipids in the form of GUVs and an initial solution concentration of 12.8 nM (3.5 pmol) of Mag2-r, to which PGLa was titrated and gently mixed. At each titration step the system was allowed to equilibrate for 15 min before each measurement. Results from the titration with unlabeled PGLa are shown in Fig. 1A and results for titration with labeled PGLa in supplementary Figure S1.

The titration experiment showed that the amount of Mag2-r bound to the POPE/POPG membrane surface depends in a linear fashion (in this regime) on the solution concentration of PGLa with a proportionality factor of (0.47 ± 0.06) × 10^–6^ m. Additionally, the amount of PGLa-g particles bound to the bilayer varied in a linear fashion with the increase in the final solution concentration of PGLa-g by a factor of (1.04 ± 0.07) × 10^–6^ m (SI Figure S1). In inverse titration experiments, the amount of PGLa-g bound to the lipid membrane was quantified for increasing amounts of magainin 2, either labeled or unlabeled (Fig. 1B). The samples consisted of approximately 16 nmol (58 µM) of lipids in the form of GUVs and an initial solution concentration of 12.8 nM (3.5 pmol) of PGLa-g, to which magainin 2 was titrated. In contrast to the behavior of magainin 2, where PGLa added to the solution helps in membrane association of magainin 2 (Fig. 1A), the amount of bound PGLa-g particles was independent on the presence of magainin 2 (Fig. 1B.).

**Figure 2:**
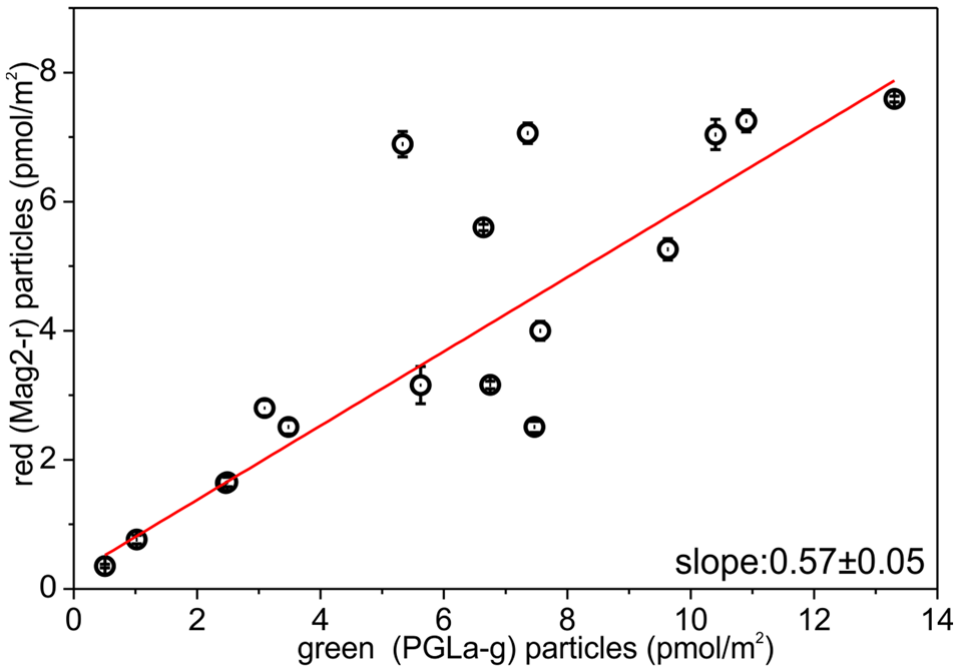
Surface concentration (pmol/m^2^) of red particles (i.e. diffusing Mag2-r particles) associated with GUVs made from POPE/POPG 2/1 mol/mol as a function of the surface concentration of bilayer-associated green particles (i.e. diffusing PGLa-g particles), during the titration of Mag2-r (concentration in solution 13 nM) with PGLa-g. Experiments were performed at 24 °C.

From the titrations performed with both labeled peptides it was possible to assess how the increase of membrane bound Mag2-r is directly related to the amount of membrane bound PGLa-g, (Fig. 2). Linear fitting of the data showed that the surface concentration of red particles (Mag2-r) on the lipid membrane was directly proportional to the surface concentration of bound green particles (PGLa-g) by a factor of 0.57 ± 0.05.

**Figure 3:**
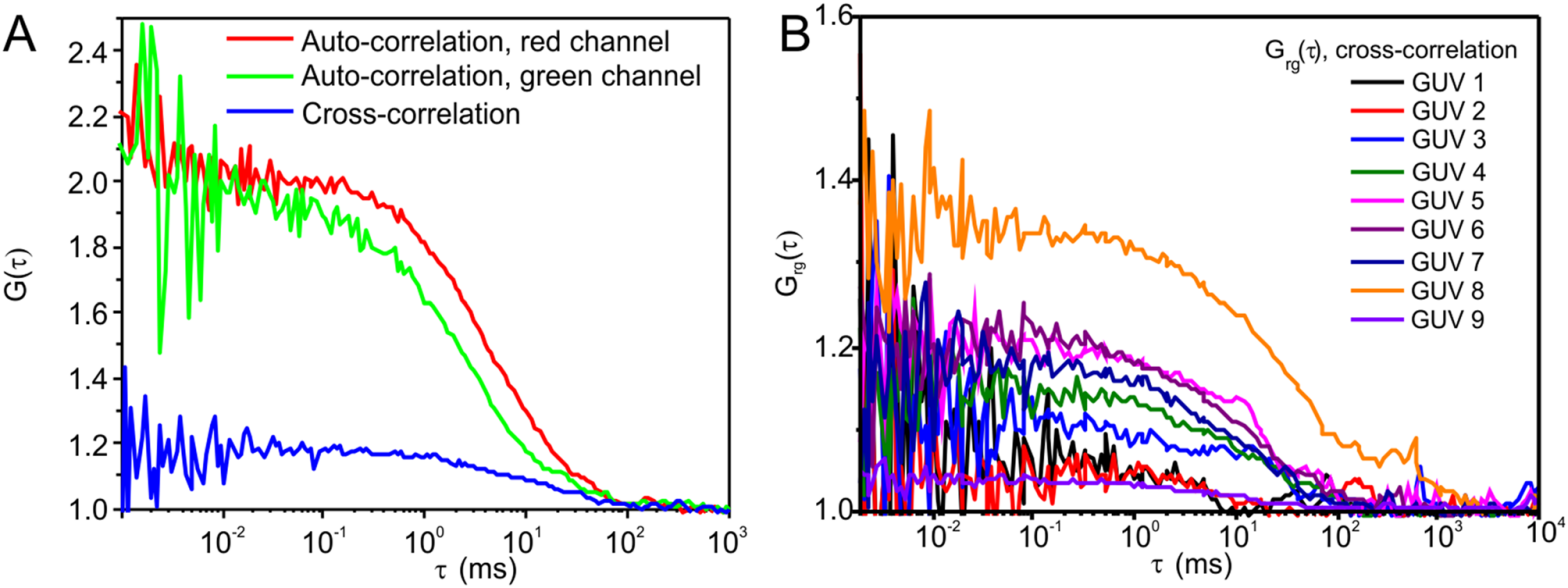
(**A**) Typical auto- and cross-correlation functions, G(τ), and (**B**) cross-correlation functions, G_rg_(τ), from 1:1 mixtures of PGLa-g and Mag2-r solutions (13 nM) in the presence of 58 µM POPE/POPG (2/1 mol/mol) in the form of GUVs. Experiments were performed at 24 °C.

Fluorescence cross-correlation spectroscopy (FCCS) experiments allow one to identify whether the two different peptides (i.e. Mag2-r and PGLa-g) form complexes or supramolecular structures. Co-diffusion of Mag2-r and PGLa-g as one entity (supramolecular structure) causes a correlation in the fluorescence signals detected in independent red and green channels, giving rise to a positive cross-correlation, that is, Grg(τ) > 1. Figure 3A shows an example of the autocorrelation functions for PGLa-g (green) and Mag2-r (red), and the cross-correlation function (blue). Figure 3B depicts only the cross-correlation functions recorded in different experiments.

The FCCS data showed that there is cross-correlation between the fluorescence signals of PGLa-g and Mag2-r (further cross-correlation results Grg(τ) at higher peptide concentrations are shown in SI Figure S2). This unequivocally shows the formation of supramolecular assemblies involving PGLa-g and Mag2-r that diffuse within the lipid membrane as one entity for a least several milliseconds.

Fluorescence self-quenching occurs when two fluorophores reside within a critical radius, thereby providing information on peptide association. In order to perform such experiments, the peptides were labeled with NBD at the amino-terminus. The fluorescence signal of magainin 2 or PGLa was recorded as a function of dilution with unlabeled peptide. Such dilutions series were recorded in the presence of increasing amounts of POPE/POPG 3/1 (mol/mol) lipid bilayers at 20 °C or 35 °C, i.e. at temperatures close to the gel-to-liquid crystalline phase transition or well above it, respectively (Tc ~ 22 °C)^27^. Finally, the titration series were repeated in the presence of the second peptide in its unlabeled form.

**Figure 4:**
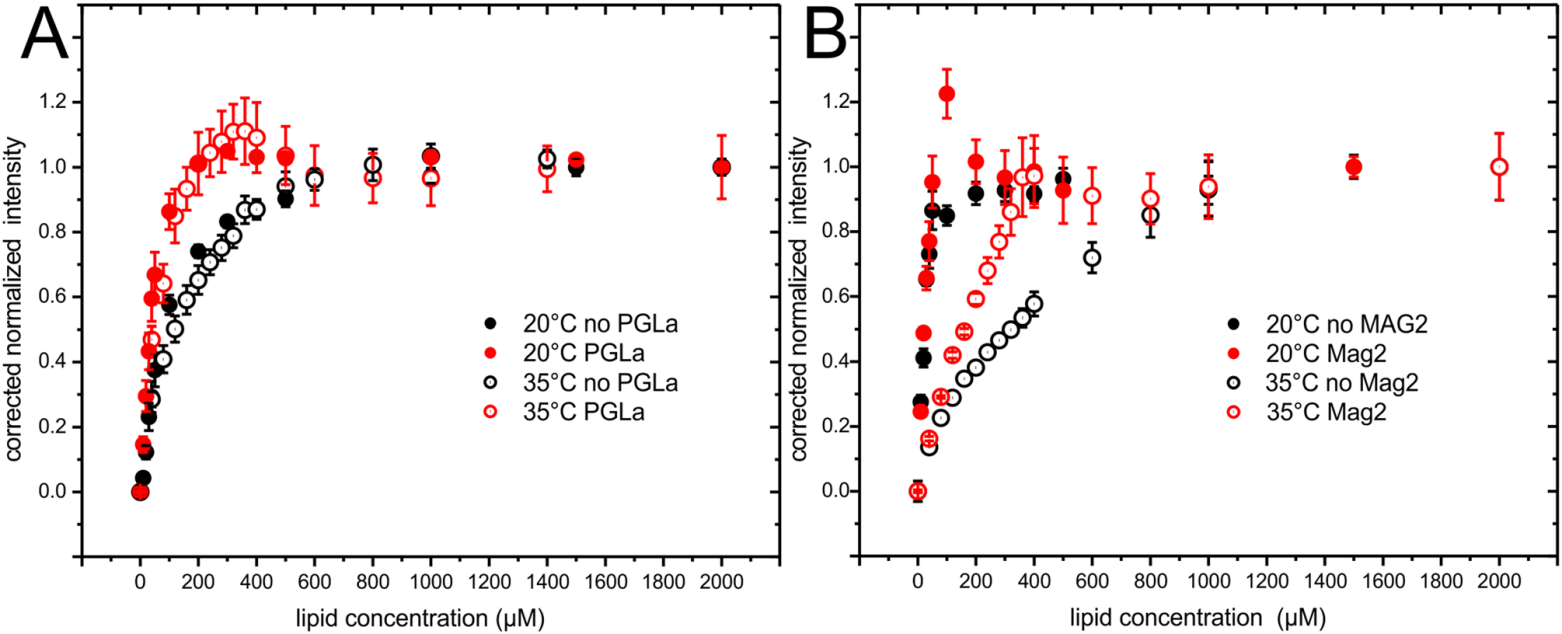
Dilution corrected and normalized intensity of (**A**) 10 µM magainin 2 in the presence (red) or absence (black) of 10 µM PGLa and (**B**) 10 µM PGLa in the presence (red) or absence (black) of 10 µM magainin 2 at 20 °C (closed symbols) or 35 °C (open symbols) for increasing lipid concentrations (POPE/POPG, 3/1 mol/mol).

**Figure 5:**
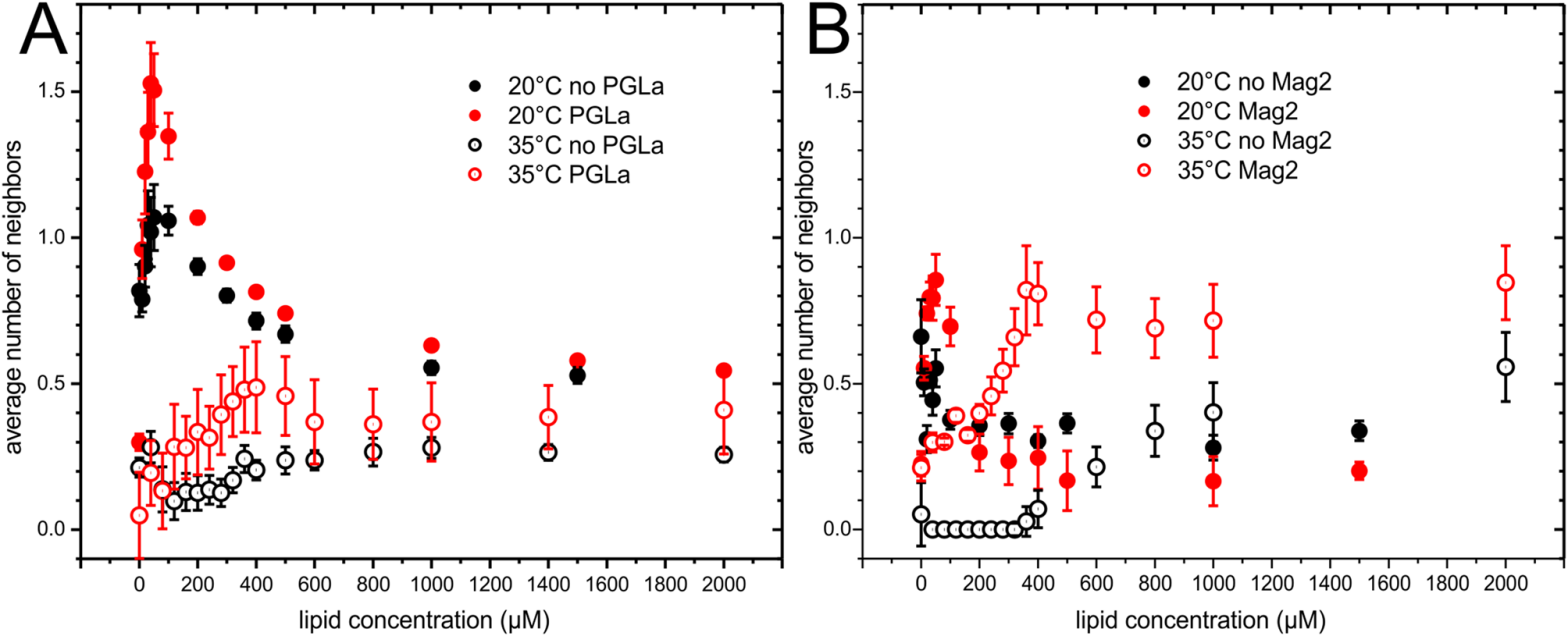
Average number of neighbors of (**A**) 10 µM magainin 2 in the presence (red symbols) or absence of 10 µM PGLa (black symbols) and (**B**) of PGLa in the presence (red symbols) or absence of magainin 2 (black symbols) for increasing amounts of lipid (POPE/POPG, 3/1 mol/mol). Measurements were performed at 20 °C (closed symbols) or 35 °C (open symbols).

The measured intensities (an example is shown in Supplementary Figure S3) were fitted with a Poisson distribution (Eq. 1) resulting in intensities that have been corrected for self-quenching (Fig. 4). Thus the number of neighbors (Fig. 5) within the self-quenching radius of about 1 nm is obtained^32^.

The self-quenching corrected intensities (Fig. 4) represent a partition isotherm^32^ and the partition coefficients were obtained using Eq. 2 (Table 1). Membrane association of magainin 2 followed a smooth function that is well described by a partition equilibrium (Eq. 2). Interestingly, the presence of PGLa increased the binding of magainin 2 at both 20 °C and 35 °C. The partition coefficients were in the range 14,000 M^−1^ (20 °C) and 3,400 M^−1^ (35 °C) in the absence of PGLa; the constants increased to 34,000 M^−1^ (20 °C) and 46,000 M^−1^ (35 °C) in the presence of PGLa.

**Table 1:** Apparent partition coefficients of the indicated peptide to POPE/POPG 3/1 (mol/mol) vesicles. The adsorption isotherms of Fig. 4 were used for the fitting.

Apparent partition coefficient (M^−1^)	20 °C	35 °C
Magainin 2 in absence of PGLa	14,050 ± 950	3,380 ± 330
Magainin 2 in presence of PGLa	34,000 ± 5,090	46,000 ± 11,000
PGLa in absence of magainin 2	> 200,000	530 ± 137
PGLa in presence of magainin 2	> 200,000	6,200 ± 3,100

The binding isotherm of PGLa was almost linear up to saturation/complete adsorption. Hence the binding seemed to be governed by limited surface area (crowding) rather than by the free energy of binding^33,34^. The curves were poorly described by Eq. 2. Nevertheless, fitting leads to values that allow for a comparison of the association with lipid bilayers. The membrane affinity of PGLa was highly dependent on the temperature. At 20 °C it was very high (partition coefficient > 200 000 M^−1^ in presence or absence of magainin 2), and barely covered by the titration protocol used here. Increasing the temperature to 35 °C significantly reduced the membrane affinity of PGLa and a partition coefficient of 530 M^−1^ was obtained. The presence of magainin 2 increased membrane association again to reach a partition coefficient in the range of 6,200 M^−1^.

**Figure 6:**
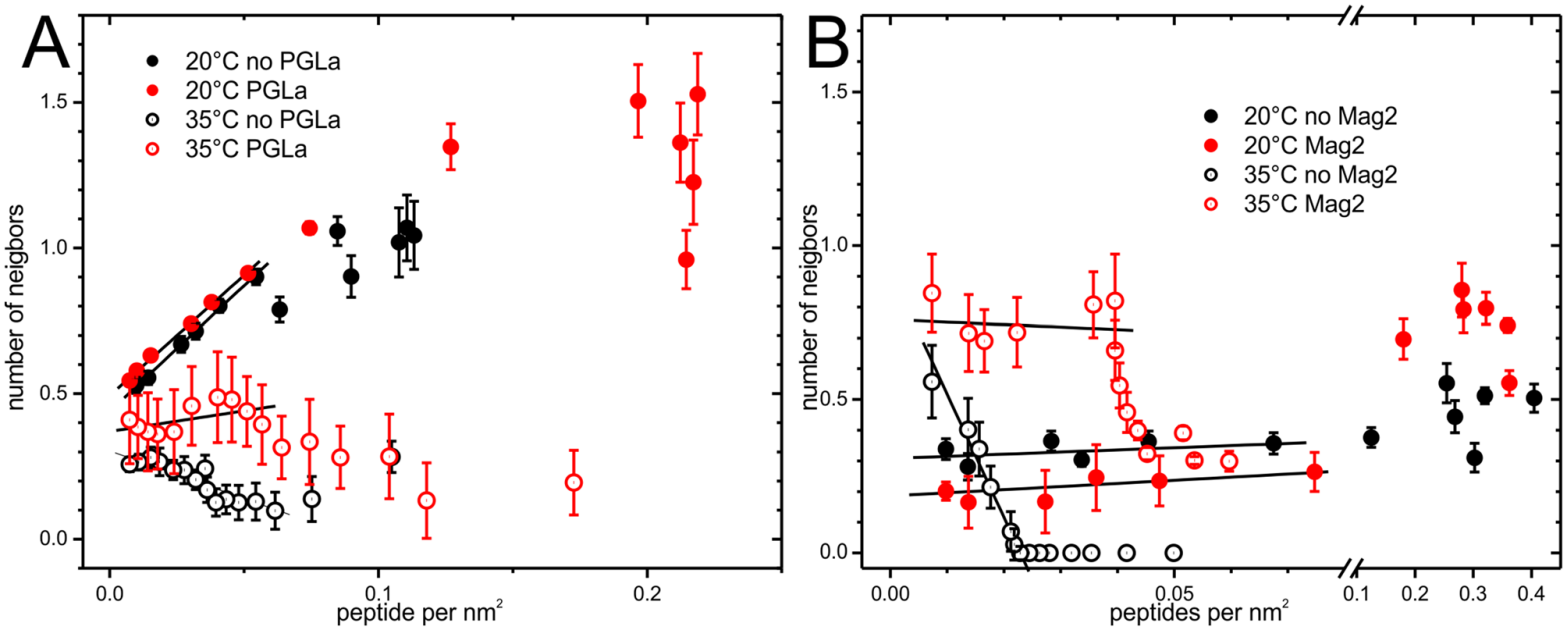
A. Average number of neighbors of 10 µM magainin 2 in presence (red symbols) or absence of 10 µM PGLa (black symbols). B. Average number of neighbors of 10 µM PGLa in presence (red symbols) or absence of 10 µM magainin 2 (black symbols). The lines correspond to linear fits below 0.05 peptides per nm^2^ of lipid surface. Measurements were performed at 20 °C (closed symbols) or 35 °C (open symbols).

The number of neighbors within the self-quenching radius of 1 nm^32^ calculated from the dilution series of the labeled peptides (Fig. 5) showed that at 20 °C the addition of lipid vesicles to magainin 2 rapidly increases the number of neighbors of magainin 2 to ~ 1.0 (Fig. 5A; closed black symbols). Above about 20 µM lipid (P/L = 1/2) the number of neighbors decreases with increasing lipid concentration in a smooth manner towards a plateau around an average of 0.5 neighbors indicating clustering even in the presence of excess lipid. Using the data presented in Fig. 5A the number of peptides per nm^2^ of lipid surface (assuming 68 Å^2^ per lipid molecule) was calculated (Fig. 6). The subsequent titration steps of increasing amounts of lipid appear from right to left in this diagram. A linear fit indicated an extrapolation of ~ 0.44 neighbors at infinite dilution at 20 °C (Table 2). The presence of equimolar amounts of PGLa results in a higher maximum number of neighbors of 1.5 at 20 µM of lipids (Fig. 5A). Analyzing the number of peptides per membrane surface shows that PGLa barely influences the packing of magainin 2 at low coverage, but allows a denser packing at higher magainin 2 coverage of the membrane surface (Fig. 6). Due to the reduced membrane affinity such high densities of magainin 2 at the membrane surface did not occur in the absence of PGLa under the conditions used in the experiments. At 35 °C the number of neighbors of magainin 2 was significantly reduced in presence and especially in the absence of PGLa. In the absence of PGLa the number of neighbors increased at lower peptide coverage (Figs. 5A, 6A), which at first glance seems paradoxal. In the presence of PGLa the number of neighbors of magainin 2 was also increased at 35 °C when compared to the absence of PGLa. Extrapolation to infinite dilution (Fig. 6A) results in 0.3 and 0.37 neighbors in the absence or presence of PGLa, respectively.

**Table 2:** Extrapolation of the number of neighbors for infinite dilutions (Fig. 6).

Number of neighbors at infinite dilution	20 °C	35 °C
Magainin 2 without PGLa	0.44 ± 0.02	0.30 ± 0.02
Magainin 2 with PGLa	0.49 ± 0.01	0.37 ± 0.08
PGLa without magainin 2	0.31 ± 0.03	0.92 ± 0.12
PGLa with magainin 2	0.19 ± 0.03	0.76 ± 0.10

The number of neighbors of PGLa on the membrane surface for increasing amounts of lipids displayed quite unique features (Fig. 5B). At low P/L ratio the presence of magainin 2 increased the number of neighbors of PGLa. However, the scattering of data made a more quantitative analysis of Fig. 5B difficult. Extrapolation to high dilution at 20 °C resulted in 0.31 (absence of magainin 2) and 0.19 neighbors (presence of magainin 2) indicating the formation of peptide clusters even when lipid is in excess. Upon increasing the temperature to 35 °C the packing of PGLa on the membrane surface dramatically changed (Figs. 5B, 6B, open symbols). Down to a P/L ratio of about 1/40 (up to a lipid concentration of 300 µM) no self-quenching could be detected indicating that the peptides were distributed in such a manner that their amino termini are at least 1 nm apart. Only at higher peptide dilution PGLa-PGLa contacts could be detected. Extrapolation lead to a number ~ 0.92 neighbors at infinite dilution (Fig. 6B, Table 2), although such linear extrapolation may exaggerate this value. The addition of magainin 2 resulted in self-quenching already at lower lipid concentrations (Figs. 5B, 6B, open red symbols), which was increased by higher dilution of the peptides on the membrane surface. The number of neighbors leveled off at ~ 0.76 for high peptide dilution.

**Figure 7:**
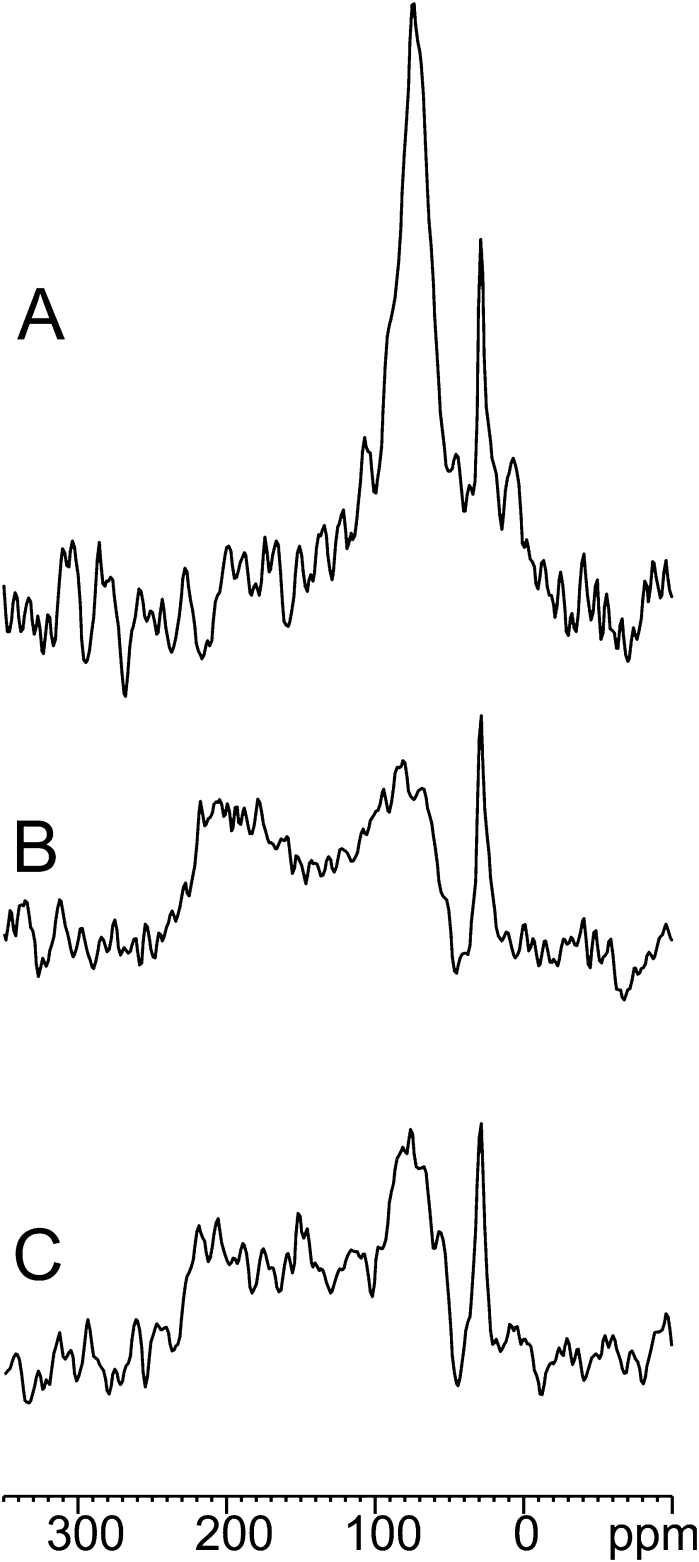
^15^N solid-state NMR spectra of [^15^N-Ala14]-PGLa (2% P/L ratio) reconstituted into POPE/POPG 3/1 (mol/mol) bilayers uniaxially oriented with the normal parallel to the magnetic field direction of the NMR spectrometer and in the presence of an equimolar amount of unlabeled magainin 2 at 35 °C (**A**), 20 °C (**B**) and 5 °C (**C**).

The unique features of PGLa in POPE/POPG 3/1 (mol/mol) at different temperatures requested additional investigations. We performed ^15^N solid-state NMR investigations of [^15^N-Ala14]-PGLa incorporated into uniaxially oriented POPE/POPG 3/1 (mol/mol) membranes (P/L = 2%) in the presence of equimolar amounts of unlabeled magainin 2 (Fig. 7). When the supported lipid bilayer sample is inserted into the NMR coil with its normal parallel to the magnetic field direction, transmembrane helical peptides exhibit ^15^N chemical shifts around 200 ppm whereas amphipathic helices oriented parallel to the membrane surface resonate < 100 ppm^35^. At 35 °C (Fig. 7A), the spectrum showed a single peak at 67 ppm indicating an in-planar alignment of the peptide. At 20 °C, the ^15^N solid state NMR spectrum (Fig. 7B) became relatively broad with contributions between 60 and 225 ppm and maxima at ~ 80 ppm and 210 ppm. This indicated the presence of many different orientations of the peptide. At 5 °C (Fig. 7C) the peak at 80 ppm became sharper. Verification of the alignment of the phospholipid bilayers by ^31^P solid-state NMR spectroscopy showed that the lipids were predominantly oriented with their long axis parallel to the magnetic field (Figure S4).

## Discussion

Fluorescence self-quenching experiments at different temperatures as well as FCS titration experiments consistently show an increased membrane affinity of magainin 2 in the presence of PGLa. The apparent partition coefficient of magainin 2 to POPE/POPG 3/1 (mol/mol) vesicles significantly increases by 2–3 fold at 20 °C and more than tenfold at 35 °C due to the presence of equimolar amounts of PGLa (Table 1). High membrane-association constants of 76 600 M^−1^ for magainin 2 and 320 000 M^−1^ for PGLa insertion into POPE/POPG 3/1 (mol/mol) have been measured before using ITC at 35 °C^36^, while CD titrations at 25 °C have suggested that the association of PGLa to this bacterial membrane mimetic is electrostatically driven and saturates in a sudden manner upon charge neutralization^37^. However, the domination of electrostatic interactions in the CD experiments is related to the low ionic strength (absence of salt) used in the experiments and can be reproduced in self-quenching experiments under the same conditions (Figure S7). Temperature has a modest effect on the affinity of magainin 2, when compared to the addition of PGLa, even though the lipids change from a liquid crystalline phase at 35 °C to a mixed liquid/gel phase at 20 °C^27^. The FCS data shows a clear linear correlation between the solution concentration of PGLa and the concentration of magainin 2 on the membrane surface (Fig. 1A). The data also suggest that two PGLa peptides are needed to help in the membrane association of one magainin 2 (proportionality factor of 0.57). Hence, the FCS data confirms that PGLa increases the membrane affinity of magainin 2. This is in line with previous findings showing that magainin exhibits a propensity to self-associate into dimers in membrane mimetic environments^38^ leading to a self-cooperative membrane absorption behavior^39^.

Notably a three to tenfold increase of the apparent association constant results in a correspondingly higher increased surface concentration of the peptides under the conditions of a typical antibiotic assay (see supplementary discussion). Assuming that the surface concentration is directly related to the bacterial killing^40^, this effect thereby accounts for an order of magnitude reduction of the MIC^7,41,42^ and can explain the synergistic behavior of both peptides under typical antibiotic assay conditions.

At 20 °C PGLa has a very high affinity to the lipid membrane, which seems to be little influenced by the presence of magainin 2 (Fig. 4B and Table 1). The increased affinity at 20 °C comes together with a change in structure from all in-planar at 35 °C to a broad distribution of peptide orientations at 20 °C (Fig. 7), while the ^31^P solid-state NMR spectra are indicative of well aligned phospholipid bilayers (supplementary Figure S4). The broad distribution of alignments of PGLa represents new membrane states which deplete the in-planar initial association state and which allow a higher packing of peptides within the membrane in the sense of molecular crowding^33^. PGLa has been documented previously to be prone to topological rearrangements in membranes made from fully saturated lipids (but not when containing palmitoyl–oleoly fatty acyl chain) as a function of the peptide-to-lipid ratio, the presence of magainin 2 and membrane hydration^14,24,25^. The disordering of the fatty acyl chain packing by molecules intercalating into the membrane interfacial region is particularly pronounced for saturated or gel state membranes and has been suggested to be a main driving force for the peptides to adopt a transmembrane state^24^.

FCCS measurements can directly track supramolecular structures that contain both peptides. FCCS experiments using PGLa-g and Mag2-r unequivocally demonstrate the presence of supramolecular arrangements that diffuse within the lipid membrane as one entity for at least several milliseconds (Fig. 3 and SI Figure S2). Fluorescence self-quenching experiments show a clear mutual influence of both peptides in the formation of supramolecular structures on the membrane surface (Fig. 6).

When the POPE/POPG membranes are in their liquid crystalline state at 35 °C, the fluorescence quenching investigations indicate that both PGLa and magainin 2 have a tendency for supramolecular arrangements at the membrane surface where their amino-termini come close to within 1 nm (Fig. 6). Previous ^15^N solid-state NMR investigations have shown that both PGLa and magainin 2 adopt stable alignments parallel to the membrane surface in liquid crystalline membranes carrying at least one unsaturation per lipid, including POPE/POPG mixtures or *E.coli* lipid extracts^24,42^. Therefore, a model emerges where a mesophase arrangement of peptide helices with head-to-head alignment brings the amino-terminal ends into close proximity, in similarity to the model previously proposed for a designed antimicrobial model peptide LAH4^32^. The group of Mazuzaki determined a magainin 2/PGLa 1:1 molar ratio as the stoichiometry where synergism is maximal^7^. A cross-linking study with peptides extended at one of their termini with a GGC tripeptide revealed a preferential reaction when the cysteines were at the C-termini of both membrane-associated peptides^43^. Here, we present data indicating that the peptides individually form a mesh-like structure which places the N-terminal fluorophores in close proximity. Notably, the average density of neighbors increases when mixed with the other peptide. Salt bridges/Coulomb/electric dipole interactions might all be important for those contacts as mutations on the C-terminal lysines of PGLa and the C-terminal glutamic acid of Mag-2 considerably reduce synergy^7,31^.

Clearly, magainin 2 has higher impact on bringing the PGLa amino-termini close to each other when compared to the influence of PGLa on magainin 2. Possibly the negative charge of the E19 residue of magainin 2 has a favorable effect on the formation of mesophase arrangements involving the heavily cationic magainin 2 (net + 3) and PGLa peptides (net + 4). Furthermore, different insertion depths of magainin 2 and PGLa^10^ may have an influence on the mesophase arrangement as the packing defects of the fatty acid chains caused by the interfacial insertion of the two peptides could partially cancel each other. The self-quenching as well as the FCS studies require that the peptides are labeled with a fluorophore which might influence the behavior of the measured peptides and thereby affects the experimental observations. However, the clear differences between the results obtained with magainin 2 or PGLa confirm the dominating effect of intrinsic peptide properties over the influence of the labels (Fig. 6). Additionally, PGLa and magainin 2 labeled with fluorophores at their amino-termini maintain their antibiotic and synergistic activity^36^ albeit with slightly higher absolute MIC values.

Additional fluorescence quenching experiments were performed at ambient temperature (20 °C), a situation likely encountered when a frog (i.e. the animal producing and storing these peptides as a cocktail) encounters a bacterial infection in a natural setting^1,2,44,45^. Because the gel-to-liquid crystalline phase transition occurs at about 21–22 °C for the POPE/POPG mixtures studied here, these two phases are expected to coexist at 20 °C^27^. At 20 °C the packing of magainin 2 is significantly denser than at 35 °C (Figs. 5A, 6A). The density further increases in the presence of PGLa (Fig. 5A) probably as a result of the increased affinity of magainin 2 to the lipid membranes in the presence of PGLa (Fig. 6A).

Bearing in mind the complex phase behavior of lipid mixtures^46^ it is not surprising that membrane peptides exhibit a distinct phase behavior in interplay with the lipids present in the system. Such systems can be described theoretically by mean field models^46^ applying the molecular theory of solutions^47^ to two dimensions. For lipid systems Yagi & Sato derived the Kirkwood–Buff (KB) integrals for radial correlation^48^ of mixtures of saturated and unsaturated lipids.

Notably, for samples where POPE is replaced by POPC (SI Figure S5) the membrane affinities of the two peptides are independent of each other. Therefore, the increased membrane affinity of magainin 2 due to the presence of PGLa is dependent on the lipid composition of the membranes, namely on the presence of POPE. Also clustering of magainin 2 and PGLa both when mixed together and when studied alone is not observed in POPC/POPG 1/3 (mol/mol) membranes (SI Figure S6). This suggests that either the H-bonding capacity of PE and/or its conical shape and resulting membrane curvature strain are important factors for the formation of peptide and possible peptide/POPE clusters. In this context it is of interest that the ^2^H-order parameters of POPE-d_31_ are considerably more affected by the peptides when compared to POPC-d_31_ when either deuterated lipid is studied in mixed membranes with POPG^24^.

In a previous investigation the synergistic activity between magainin 2 and PGLa was related to the negative curvature strain imposed on the lipid bilayers due to the presence of POPE lipids, with PGLa possibly being able to precondition the membrane for magainin 2 action^26^. Thus, lipid structure and packing play important roles in the interaction of both peptides with the membrane and with each other. Importantly, the features of POPE may provide a mechanism for the selective activity of the peptides, since lipids with intrinsic negative curvature, such as PE, are enriched in *E. coli* and other bacterial membranes^49^. In addition, the selective killing of bacteria is assured by the strong cationic character of the peptides which increases their local concentration in the proximity of the negatively charged bacterial surface^50,51^. Lipid-dependent activities of antibiotic peptides have been described before^17,26,27,52,53^ featuring an “active” role of lipids in the antimicrobial action^22^.

## Conclusion

Both PGLa and magainin 2 form clusters on the membrane surface specifically in the presence of POPE, a lipid with a negative intrinsic curvature^27^ typical for bacterial membranes. The peptides’ selective action against bacteria is assured not only by the negative surface charge of the bacterial outer membrane that increases the local concentration of the cationic peptides but also by their clustering in the presence of PE-rich membranes as described here. Additionally, our experimental results demonstrate the mutual influence of both peptides in the formation of even tighter macromolecular assemblies. Thereby, the lateral organization of peptides adds an additional layer of complexity to their mechanism of action. Peptide-rich domains (the hallmark of the carpet model^54^) probably modify the lipid structure including the lipid fatty acid chain packing^55^ and the bilayer integrity, as described in the SMART model^22^. Interestingly, the mere presence of peptide-induced domains and the concomitant domain boundaries may be responsible for increased membrane permeabilization^55–58^. Most strikingly, when PGLa and magainin 2 are added together to POPE/POPG membranes their affinity to this bacterial membrane mimetic is considerably increased, which by itself can explain the synergistic enhancement of antibiotic activities observed for the combination of peptides. Notably, the thermodynamic characteristics are tightly related to the structural features of the formation of mesophases.

## Methods

### Peptide synthesis

The peptides were synthesized as described in^18^ following the standard Fmoc-strategy using an automatic Milipore 9,050 peptide synthesizer (Millipore cooperation, Bedford, MA, USA) and Fmoc-protected amino acids (Novabiochem/Merck, Nottingham, UK). The peptide sequences are:

- magainin 2 (Mag 2): GIGKF LHSAK KFGKA FVGEI MNS-carboxyamide.
- PGLa: GMASK AGAIA GKI{^15^N-A}K VALKA L-carboxyamide.

### NBD-labeling

The labeling of the peptides with NBD was performed when still attached to the resin and the side chains protected. Therefore, the N-terminus represented the only free amine after cleavage of the final Fmoc protection group. 10 equivalents of 4-Chloro-7-nitrobenzofurazan (NBD) (Fluka, Sigma-Aldrich, St. Louis, Missouri, USA) was dissolved in 50% acetonitrile containing 50 mM NaHCO_3_ and the resin was incubated for 8 h (typically 2 ml/100 mg resin)^59^. After cleavage from the resin the peptides were purified by reverse phase HPLC. The identity and purity of the products were confirmed by MALDI mass spectrometry.

### Bodipy labeling

5 mg of peptide was dissolved in 1 ml of 50% acetonitrile and the pH was adjusted to 6 by drop-wise additions of 100 mM NaOH. 0.5 mg of activated BODIPY (FluoProbes FL SE, Interchim, Montlucon, France for PGLa and BODIPY 650/655-X, SE Molecular Probes, Eugene, Oregone, US for Mag2) was dissolved in 300 µl of 50% acetonitrile. Both solutions were mixed and incubated at 4 °C over night. The product was purified by HPLC and identified by mass spectrometry. The attachment to the N-terminus was ensured by the relatively low pH value well below the pK of the lysines. In the case of PGLa the attachment of the label to the N-terminus was verified by NMR by using a ^15^N labeled glycine at the N-terminus.

### Self-quenching fluorescence measurements

Fluorescence measurements were performed in 10 mM Tris pH 7, 1 mM EDTA and 100 mM NaCl. Fluorescence spectroscopy was performed using a FluoroLog spectrophotometer (HORIBA, Ltd., Kyoto, Japan) with the polarization filters at the magic angle. The slit size was 3 nm for excitation and emission. For NBD fluorescence the emission was scanned from 470 to 650 nm at an excitation wavelength of 465 nm^32^.

### Self-quenching analysis

For every series of measurements, the label was diluted by replacement of the labeled peptide with unlabeled peptides. The resulting series contained 100%, 66.7%, 50%, 33.3% and 20% (per mole) of labeled analogues. The intensities at 540 nm for the series were fitted assuming a Poisson distribution of the number of neighbors^32^:

1 $$I(I_{0} ,\lambda ,\delta ) = I_{0} e^{ - \delta \,\lambda }$$

where I_0_ corresponds to the intensity in absence of self-quenching, $\delta$ is the average number of neighbors and $\lambda$ is the fraction of labeling.

Fitting of apparent partition equilibrium:

Partition isotherms were fitted with the equation:

2 $$I\left( {\left[ {lip} \right]} \right) = I_{0} + \Delta I\frac{1}{{1 + {\raise0.5ex\hbox{$\scriptstyle 1$} \kern-0.1em/\kern-0.15em \lower0.25ex\hbox{$\scriptstyle {k\left[ {lip} \right]}$}}}}$$

The equation follows directly from the relations:

$$k = \frac{{\left[ {pep_{bound} } \right]}}{{\left[ {lip} \right]\left[ {pep_{free} } \right]}},\left[ {pep_{free} } \right] + \left[ {pep_{bound} } \right] = \left[ {pep} \right]\;{\text{and}}\;I = I_{f} \frac{{\left[ {pep_{free} } \right]}}{{\left[ {pep} \right]}} + \left( {I_{f} + \Delta I} \right)\frac{{\left[ {pep_{bound} } \right]}}{{\left[ {pep} \right]}}$$

where k is the equilibrium constant, [pep_bound_], [pep_free_], [pep], are the concentration of bound , free and total peptide respectively.[lip] is the concentration of lipids, I_f_ is the signal of the free peptide and ΔI is the intensity change due to binding.

The equations are formally identical to a binding in the sense of the mass action law. The interpretation of

$$c_{lip}^{pep} = \frac{{\left[ {pep_{bound} } \right]}}{{\left[ {lip} \right]}}$$

as the concentration of the peptide within the membrane allows to read the equations as a partition equilibrium.

## Preparation of small unilamellar vesicles:

POPC, 1-palmitoyl-2-oleoyl-sn-glycero-3-phosphocholine (Sigma-Aldrich, St. Louis, Missouri, USA), POPE, 1-palmitoyl-2-oleoyl-sn-glycero-3-phosphoethanolamine (Avanti Polar Lipids, Alabaster, Alabama, USA) and POPG, 1-palmitoyl-2-oleoyl-sn-glycero-3-phospho-(1′-rac-glycerol) (Avanti Polar Lipids, Alabaster, Alabama, USA) were co-dissolved in dichloromethane in a small test tube and the solvent was evaporated under a stream of nitrogen. The remaining traces of organic solvent were removed by high vacuum overnight. The appropriate amount of 10 mM Tris pH 7, 1 mM EDTA and 100 mM NaCl was added in order to obtain a 10 mM lipid suspension. The samples were first vortexed and then homogenized by four freeze–thaw cycles. Small unilamellar vesicles were produced by extrusion 21 times through a poly-carbonate film of 100 nm using a syringe extruder (Avanti Polar Lipids, Alabaster, Alabama, USA)^60^.

### Preparation of Giant unilamellar vesicles by gentle hydration

POPE and POPG (2/1 mol/mol) were mixed in 1 mL chloroform (approx. 0.28 mg lipid/mL). The solution was dried with a rotary evaporator to form a lipid film and then kept under vacuum for 6 h to eliminate all traces of the solvent. The dried film was hydrated with 1 mL of pre-warmed buffer (10 mM Tris, 100 mM NaCl, 0.1 M sucrose, pH = 7.1) that had been purged with nitrogen^61^. The sample was then sealed, heated to 37 °C, kept overnight at this temperature and then slowly cooled. The resulting suspension was gently vortexed before further use to disperse the formed liposomes. The prepared lipid mixtures contained 2 mol% of biotinyl-Cap-PE necessary for immobilization of the giant unilamellar vesicles (GUVs) to BSA-biotin/streptavidin-coated microscopy chambers. To prepare a sample for measurement, 40 µL of the GUVs suspension were placed on a microscopy chamber containing 200 µL of glucose buffer (10 mM Tris pH 7, 100 mM NaCl, 0.1 M glucose). After inspection of the GUVs quality, PGLa or/and magainin 2 were added to the chamber at a final concentration of 13 nM each, unless otherwise stated. Magainin 2-Bdp650 (Mag2-r) and PGLa-FL (PGLa-g) are used considering 100% labeling and at a 1:1 ratio (unless otherwise stated). Experiments were performed at 24 °C.

### Two-color fluorescence correlation spectroscopy (FCS) measurements

Measurements were performed on a home-built confocal microscope consisting of an inverted confocal microscope body IX71 (Olympus, Hamburg, Germany). Pulsed diode lasers (LDH-P–C-470, 470 nm, and LDH-D-C-635, 635 nm; PicoQuant, Berlin, Germany) were used at 10 MHz repetition rate each and pulsed alternatively to eliminate cross-talk. Laser light was coupled to a polarization maintaining single mode optical fiber and re-collimated at the output with an air space objective (UPLSAPO 4X). The light was up-reflected with a 470/635 dichroic mirror onto a water immersion objective (UPLSAPO 60x, Olympus). The fluorescence signal was split between two single photon avalanche diodes using 515/50 and 697/58 band pass filters (Chroma Rockingham, VT) for the green and red channels, respectively. Laser intensity at the back aperture of the objective was approximately 10 µW for each laser. Measurements were performed on the top of selected GUVs. For z-scan FCS^62^, the membrane was first placed on the waist of the focus of the laser and then moved 1.4 µm above it. A sequential vertical scan of 20 steps (spaced 150 nm) was then performed, where at each z-position a measurement was taken for 60 s. For fluorescence cross-correlation spectroscopy (FCCS) measurements, the membrane was placed on the waist of the focus and data was acquired for a period of 5 min on each GUV.

### Analysis of the FCS data

The auto-correlation function G(τ) was calculated from the fluorescence intensity traces in each independent channel (red or green) and fitted to a model that includes two- and three-dimensional diffusions, which allows to account for a fraction of unbound peptide remaining in solution^63^:

3 $$G(\tau ) = 1 + \frac{1}{N(1 - T)}\left[ {1 - T + Texp\left( {\frac{ - \tau }{{\tau_{T} }}} \right)} \right] \times \left[ {\frac{{1 - F_{2} }}{{1 + \left( {\frac{\tau }{{\tau_{D3} }}} \right)}}\frac{1}{{\left[ {1 + \left( {\frac{\tau }{{\tau_{D3} }}} \right)\left( {\frac{{\omega_{0} }}{{\omega_{z} }}} \right)^{2} } \right]^{1/2} }} + \frac{{F_{2} }}{{1 + \left( {\frac{\tau }{{\tau_{D2} }}} \right)}}} \right]$$

where *τ*_*D*2_ and *τ*_*D*3_ are the diffusion times of molecules diffusing in two and three dimensions respectively, *ω*_0_ is the waist of the focus (where the intensity has dropped by 1/e^2^), *ω*_*Z*_ is the characteristic axial dimension of the detection volume, *T* is the average fraction of fluorophores in triplet state and *τ*_*T*_ the intersystem crossing relaxation time. *F*_2_ is the fraction of molecules diffusing in the two-dimensional space, i.e. membrane bound and laterally diffusing. *τ*_*D*2_ and *τ*_*D*3_ are the diffusion times of the molecules moving in two and three dimensions, respectively. *τ*_*D*3_ was determined by measurements in bulk solution.

Fitting of each auto-correlation function obtained during the z-scan retrieves the τ_D2_, particle numbers N and the fraction of membrane-associated molecules. The surface concentrations *C* of the labeled peptides were determined by fitting the plot of *N* against the focus position z to a parabolic dependency, as detailed in Benda et al.^62^:

4 $$N = \pi C\omega_{0}^{2} \left( {1 + \frac{{\lambda^{2} \Delta z^{2} }}{{\pi^{2} n^{2} \omega_{0}^{4} }}} \right)$$

where *n* is the refractive index of the medium, *λ* is the wavelength of the excitation light, and ∆*z* is the distance between the sample position *z*_0_ and the position of the beam diameter minimum *z.*

The cross-correlation function G_rg_(τ) was calculated from the intensity traces in the red and green channels.

### Uniaxially oriented samples for solid state NMR spectroscopy

37 mg of POPE and 13 mg of POPG (POPE/POPG 3/1 mol/mol) were co-dissolved with 0.7 mg [^15^N-Ala14]-PGLa (2%) and 0.8 mg of magainin 2 (2%) in chloroform methanol 3/1. All peptides were used without any fluorescence label attached. The volume was reduced with a stream of nitrogen and the solution distributed on 20 ultrathin cover plates (13 × 8 mm) (Marienfeld GmbH, Lauda-Königshofen, Germany). The samples were first dried on air and then overnight in high vacuum. After rehydration in 93% humidity the glass plates were stacked, the stack was stabilized by Teflon tape and sealed in Plastic (Escal neo, Mitsubishi Gas Chemical Company Inc. Tokio, Japan).

### Solid-state NMR spectroscopy

Proton decoupled ^15^N spectra were recorded using a simple cross-polarization experiment on a 750 MHz wide bore NMR spectrometer (Bruker Biospin, Karlsruhe, Germany) with a B1 field of 50 kHz for both the proton Pi pulse and the CP step of 400 µs. Typically 20,000 scans were accumulated. Proton decoupled ^31^P spectra were recorded on a 300 MHz wide bore NMR spectrometer (Bruker, Karlsruhe Germany) using a hahn echo experiment of 10 µs echo time. The ^31^P B1 fields were 83 kHz and 64 scans were accumulated.

### Mag2-r titrated with PGLa-g

Mag2-r (3.5 pmol, solution concentration of 12.8 nM) was added to samples containing 16 nmol of lipids as GUVs (58 µM). This sample was then titrated with PGLa-g. The last sample corresponds to the same conditions used for samples A of the FCCS measurements, 13 nM of each peptide and 58 µM of lipids. The measurements are made in two-colour mode, i.e., data is obtained simultaneously for both peptides, for each particular GUV.

## Supplementary information

Supplementary Information.
