# Supplementary material for: Highly synergistic antimicrobial activity of magainin 2 and PGLa peptides is rooted in the formation of supramolecular complexes with lipids
Source: Sci Rep. 2020 Jul 15;10:11652. doi: 10.1038/s41598-020-68416-1 (PMC7363891; doi:10.1038/s41598-020-68416-1)
Supplement: Supplementary file 1 — Supplementary Information. [file 41598_2020_68416_MOESM1_ESM.docx]

**Supplementary Information:**

**Highly synergistic antimicrobial activity of Magainin 2 and PGLa peptides is rooted in the formation of supramolecular complexes with lipids**

Christopher Aisenbrey1,*, Mariana Amaro2,*, Petr Pospíšil2,^†^, Martin Hof2 and Burkhard Bechinger1,3

^1^ Institut de Chimie UMR7177,CNRS, University of Strasbourg, 1, rue Blaise Pascal, 67000 Strasbourg, France.

^2^ J. Heyrovský Institute of Physical Chemistry,v.v.i. Czech Academy of Sciences, Dolejškova 2155/3, 182 23 Prague, Czech Republic

^3^ Institut Universitaire de France, Paris, France.

[*aisenbrey@unistra.fr](mailto:*aisenbrey@unistra.fr) and [mariana.amaro@jh-inst.cas.cz](mailto:mariana.amaro@jh-inst.cas.cz)

^†^ present address: Institut für Angewandte Physik and Center for Functional Nanostructures (CFN),Karlsruhe Institute of Technology, Wolfgang-Gaede-Straße 1, D-76131 Karlsruhe.

**supplementary Figure S1:** Surface concentration (pmol/m^2^) of bilayer-associated particles as a function of the final concentration (nM) of PGLa-g in solution (i.e. diffusing Mag2-r particles or PGLa-g particles). Mag2-r concentration in solution is (aprox.) constant at 13 nM.

**Supplementary Figure S2:** Cross-correlation functions (G_rg_(τ)) obtained using a 1:1 mixture of PGLa-g and Mag2-r solutions and 58μM of POPE/POPG (2/1) lipids in the form of GUVs. [PGLa-g]= [Mag2-r]= 18nM


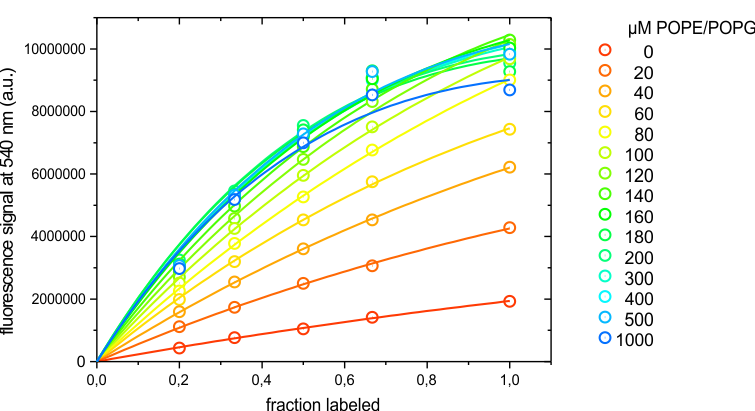


**Supplementary Figure S3:** Fluorescence signal at 540 nm of 10µM of PGLa (partly or completely labeled with NBD) in the presence of 10µM of unlabeled Mag 2 at 35°C at increasing concentration of POPE/POPG 3/1 vesicles,


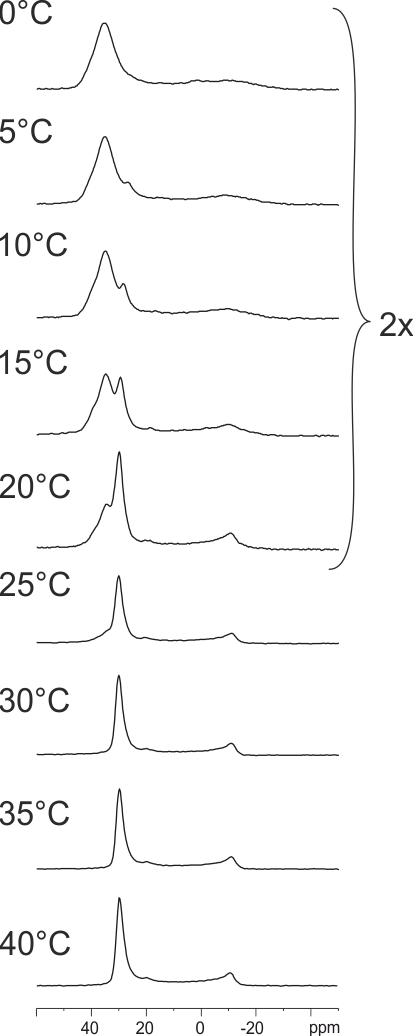


**Supplementary Figure S4:** Proton decoupled ^31^P solid-state NMR spectra of oriented POPE/POPG samples in the presence of 2% PGLa and 2% magainin 2. The intensity of the spectra recorded at 0^o^ to 20^o^ is 2x increased.


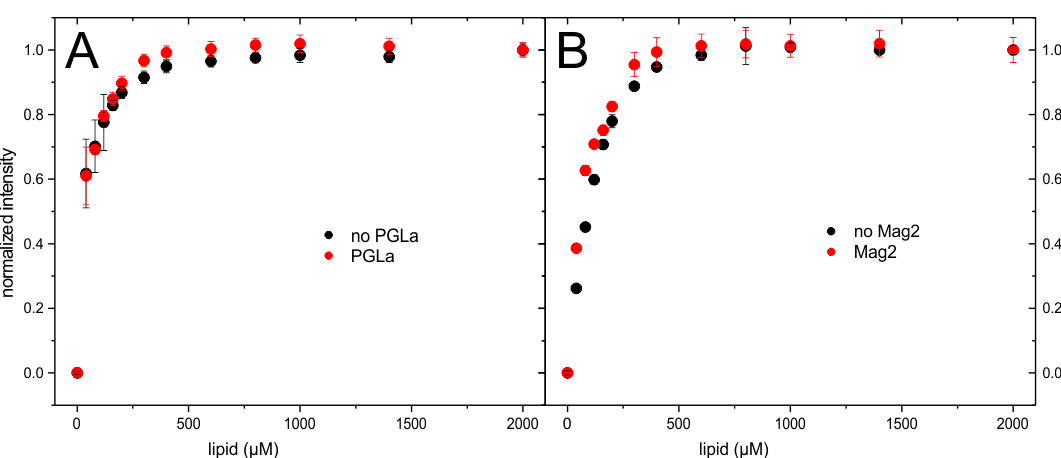


**Supplementary Figure S5:** Self-quenching corrected intensities of the titration of 10µM of Mag2 (A) and 10µM of PGLa with POPC/POPG 3/1 in the absence or presence of equimolar amounts of the other peptide at 20°C. Dissociation constants are: magainin 2 (no PGLa): 34000M^-1^, magainin (with PGLa) ; 37000M^-1^, PGLa (no magainin 2): 14500 M^-1^ PGLa (with magainin 2) 19550M^-1^.


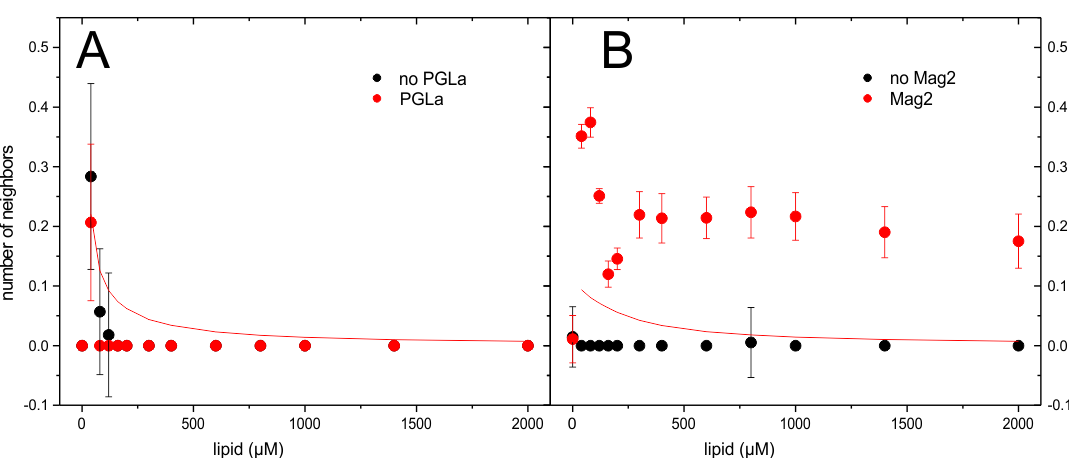


**Supplementary Figure S6:** Number of neighbors obtained from the titrations of 10µM of Mag2 (A) and 10µM of PGLa with POPC/POPG 3/1 in the absence or presence of equimolar amounts of the other peptide at 20°C.


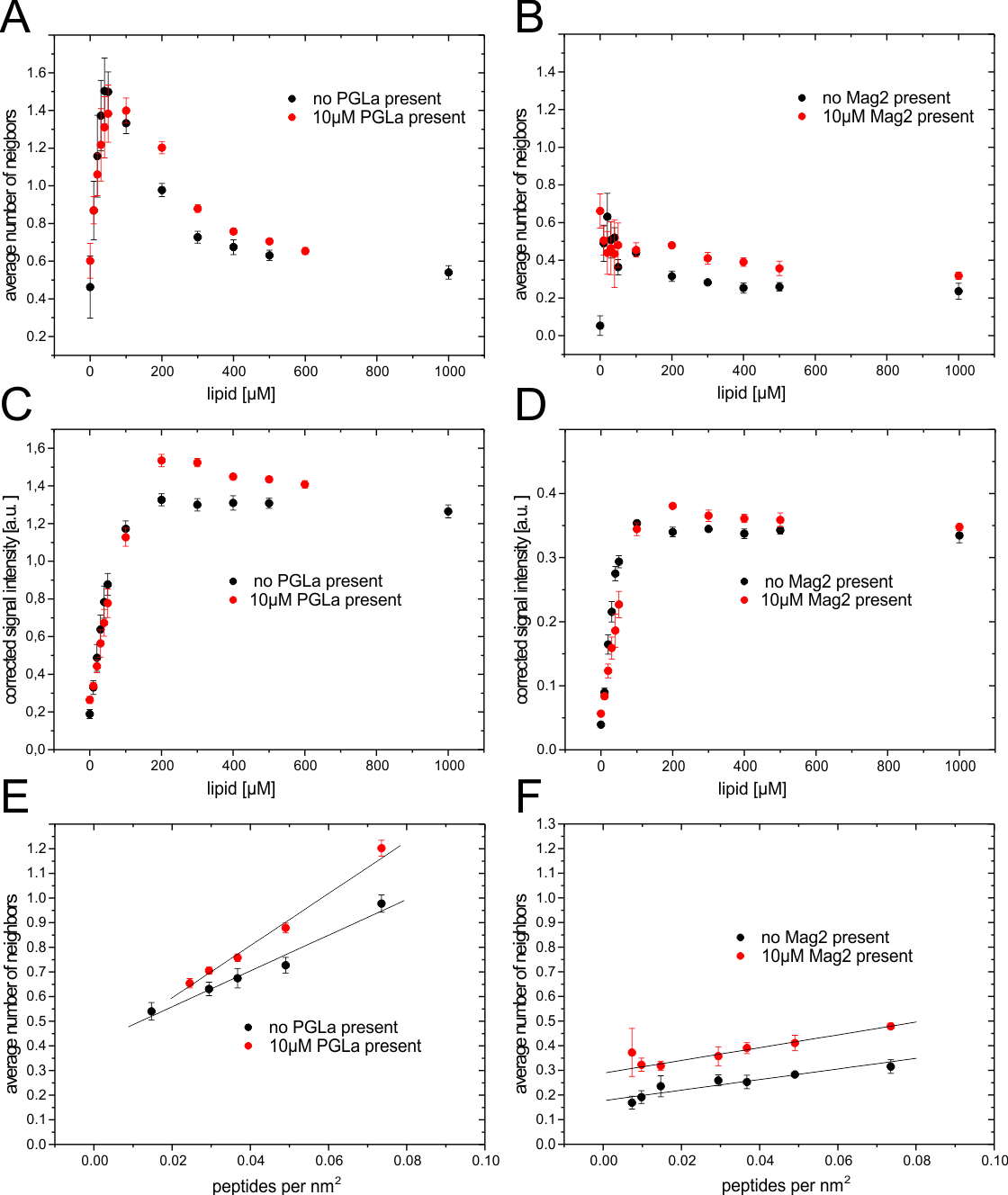


**Supplementary Figure S7:** Lipid titration experiments (POPE/POPG 3/1 mol/mol) of 10µM NBD labeled (partially) Mag2 (A,C,E) and 10µM NBD labeled (partially) PGLa (B,D,F) in the presence of 10 mM Tris in absence of additional salt. The red symbols represent measurements in the presence of equimolar amounts of the second peptide. A and B are the number of neighbors for increasing lipid concentrations. C and D are the self-quenching corrected intensities for increasing lipid concentrations, E and F are the average number of neighbors as a function of peptide density on the membrane surface.

**Supplementary discussion: Effect of association constant on the MIC:**

$k=\frac{\left[pep_{bound}\right]}{\left[lip\right]\left[pep_{free}\right]}$ $\left[pep_{free}\right]+\left[pep_{bound}\right]=\left[pep\right]$

Leads to:

$$\frac{\left[pep_{bound}\right]}{\left[pep\right]}=\frac{k\left[lip\right]}{1+k\left[lip\right]}\approx k\left[lip\right] for \left[lip\right]<<\frac{1}{k}$$

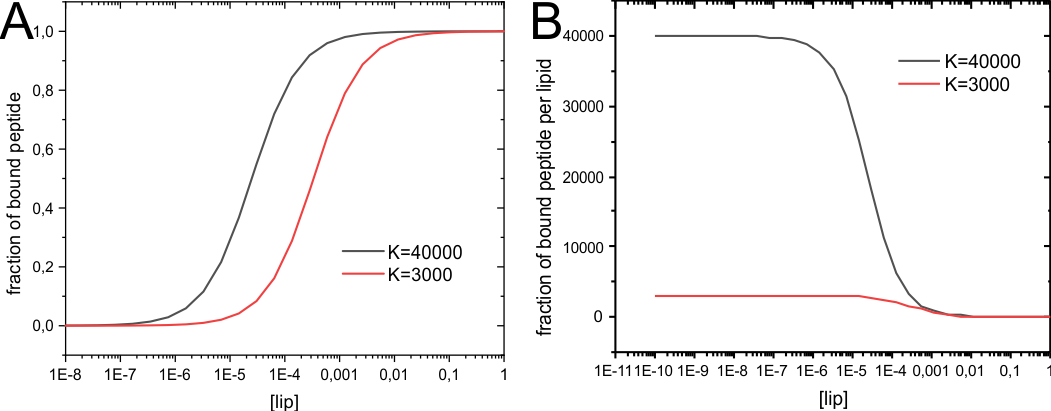


**Supplementary Figure S8:** Graphical representation of the fraction of bound peptide (A) and fraction of bound peptide per lipid concentration (B) for K=3000 (red) and K= 40000 (black)

Hence, for $\left[lip\right]<<\frac{1}{k}$ the fraction bound peptide per lipid is directly proportional to the association constant and the MIC is inversely proportional to the association constant since it is related to the bulk concentration of the peptide.

Estimation of [lip] in a bacterial test:

20000 E. coli per ml (typical experimental parameter)

E. coli dimensions: 0.5 µm x 2 µm

0.6 nm^2^ surface area per lipid molecule

Surface area per E. coli: ~4µm^2^

Number of surface lipids per E. coli:

~14 *10^6^ surface lipids per e-coli (both leaflets)

Lipids per volume for 20000 E. coli per ml:

2.8 10^14^ lipids per liter -> 4.6 10^-10^ mol surface lipids per liter -> in the nM range well below 1/K
